# 

**Published:** 1859-03

**Authors:** 


### CONTENTS

OF THE

### Chicago Medical Journal---March, 1859.

ORIGINAL COMMUNICATIONS.

PAGE.

ART. I. Case of a Foreign Body in the Air Passages Three Months. Successful Operation; with suggestions on the best means of preventing Hemorrhage during the operation, and obviating the effects of cold air after Tracheotomy. By Daniel Brainard, M.D., Surgeon of the U. S. Marine Hospital, Chicago........ 133

ART. II. Stomatitis Materna: Report of Committee on Practical Medicine of Ill. State Medical Society for 1858: a Correction, etc. By J. N. Green, M.D., of Stilesville, Indiana........ 137

ART. III. Cases illustrating the Practical Use of the Ophthalmoscope, as a means of Diagnosis, in certain Diseases of the Eye. By E. L. Holmes, M.D., of Chicago........ 139

ART. IV. The Alkaline Treatment of Rheumatism. By B. F. Schneck, M.D , of Lebanon, Pa........ 142

ART. V. Spongy Gums. By T. W. Fry, M.D., of Crawfordsville, Ind..... 149

ART. VI. Intestinal Concretions. By F. K. Bailey, M.D., of Joliet, Ill... 150

ART. VII. Injection of Solution of Nitrate Silver into the Larynx in Membranous Croup. By J. H. Reeder, M D., of Lacon, Ill........ 154

ART. VIII. Obstruction of the Bowels, and Extra-Uterine Pregnancy. By Dr. R. F. Henry, of Princeville, Ill........ 155

ART. IX. Case of Cancer of the Conjunctiva. Extirpation of the Globe of the Eye. Case of Nasal Polypus, cured by operation and applications. By Charles Brackett, M.D., of Rochester, Ind........ 158

EXTRACTS.

An Address to the Graduates of the Ohio College of Dental Surgery, session of 1858-9, by W. W. Allport, D.D.S., of Chicago........ 160

Straightening a Limb by Dr. Brainard's Method. By Paul F. Eve, M.D... 161

Anchylosis of the Knee Joint. Novel Mode of Treatment by Fracture of the Thigh Bone........ 162

ABSTRACTS AND ITEMS.

BY W. H. BYFORD, M.D., OF CHICAGO. 165 to 169

BOOK AND PAMPHLET NOTICES.

A Treatise on Fractures. By J. F. Malgaigne. Translated from the French, by J. H. Packard, M.D........ 170

Mind and Matter. By Sir Benj. Brodie, Bart., D.C.L........ 170

The Uremic Convulsions of Pregnancy, Parturition and Childbed. By Dr. Carl L. Braun, Vienna. Translated from the German, by S. M. Duncan, F.R.C.P.E........ 171

Of Nature and Art in the Cure of Disease. By Sir John Forbes, M.D...... 175

New Journals........ 175

TRANSLATIONS BY DR. E. L. HOLMES.

Influence of different beverages upon the elimination of common Salt, Urea and Sugar in Diabetic Urine........ 177

Ætiology of Parenchymatous Nephritis........ 178

A New Apparatus for the Extension of Flexed Knee Joint........ 180

EDITORIAL.

Dr. Davis' Valedictory; Dr. Brainard's Salutatory........ 183

Rush Medical College........ 185

MISCELLANEOUS........186 to 192

